# PGE1 nebulisation during caesarean section for Eisenmenger's syndrome: a case report

**DOI:** 10.1186/1752-1947-2-149

**Published:** 2008-05-09

**Authors:** Shahla Siddiqui, Naveed Latif

**Affiliations:** 1 Department of Anaesthesia, Aga Khan University, Karachi, Pakistan

## Abstract

**Introduction:**

Eisenmenger's syndrome in pregnancy can lead to death in 50% to 65% of parturients. Expensive invasive monitoring and medication have improved management and outcomes. Cheaper alternatives for the management of high-risk patients who present with no prenatal care are still not available.

**Case presentation:**

We describe the obstetric anaesthesia management of a 34-year-old, 34-weeks pregnant woman who presented with a recent diagnosis of severe Eisenmenger's syndrome. A combined spinal epidural anaesthesia was used together with invasive cardiac monitoring as well as PGE1 nebulisation after delivery of the baby. This helped achieve a reduction of shunt, improvement of hypoxia and reduction of pulmonary pressures.

**Conclusion:**

We found this to be a cheaper and safe alternative in the management of such patients who present with no adequate prior management.

## Introduction

Eisenmenger's syndrome with pulmonary hypertension and reversal of shunt can be a lethal condition when combined with pregnancy. Chronic hypoxia, dyspnoea and orthopnoea with severely diminished exercise tolerance as well as right heart failure is often the pathology involved with deterioration [1]. As pregnancy progresses the pulmonary gradient worsens and shunt reversal increases. Early literature seems to suggest that termination of pregnancy is the only option to save the parturient's life [2]. However, the recent literature reports successful pregnancy and delivery when the patient is managed with various modes including sildenafil and prostacyclin from early gestation [3,4]. However, these are high-risk cases that are managed from the first trimester onwards with oral medication. There is, as yet, no description of patients who present for delivery at full term with no management with pulmonary antihypertensive agents and with a critical pulmonary gradient.

## Case presentation

We present the anaesthetic management of a 34-year-old gravida III, para 1 woman who was 34 weeks pregnant and who presented to a University Hospital setting for caesarean delivery. She was recently diagnosed with pulmonary arterial hypertension (PAH) and atrial septal defect (ASD). Her foetus was also diagnosed on a prenatal ultrasound as having an ASD. She was also suffering from gestational diabetes. Her clinical history included dyspnoea at rest and orthopnoea requiring two pillows whilst sleeping. She had a severely restricted functional capacity. She had a previous uneventful caesarean section 4 years prior as well as a miscarriage in the first trimester 3 years prior. She admitted that her symptoms were much worse in this pregnancy. Her physical examination revealed a pansystolic murmur in the left apex. An echocardiogram revealed a moderate sized ASD with severe PAH, moderate pulmonary and tricuspid regurgitation and enlarged right heart. A cardiology consultation recommended placing her on oral furosemide, aspirin and potassium supplements.

After counselling the patient and her family pre-operatively in consultation with the obstetrician, the patient was advised regional anaesthesia for the delivery. On the day of the caesarean delivery, after routine monitors were placed in the operating room, a radial arterial line was placed for beat-to-beat blood pressure monitoring. Her baseline saturation as revealed by the use of a pulse oximeter was 89% to 95% on facemask oxygen. She was then placed in a sitting position where a combined spinal epidural technique was used to deliver a slow graded spinal with 1 cc 0.5% bupivacaine and 20 mcg of fentanyl with a 27-gauge spinal needle. She was then placed in a semi-sitting position at 45° propped up on pillows as much as her orthopnoea would allow. Another 2 cc of 0.25% bupivacaine was administered via the epidural catheter to establish a block up to T4 level. A strong motor block was accomplished. Systemic blood pressures were supported by boluses of phenylephrine. Caesarean section was then performed without delay in the semi-recumbent position without event and the patient remained comfortable. A pulmonary artery (PA) catheter was floated during surgery to reveal PA pressures of 60 to 70 systolic. After delivery of the baby, the patient was nebulised with 1 cc of prostacycline (PGE1) and 4 cc of normal saline via a facemask for 30 minutes. Her arterial saturations improved to 99% and PA pressures reduced to 30 to 40 systolic. Systemic blood pressures were stable. The entire procedure lasted 1.2 hours with no untoward event and both mother and baby remained well post-operatively. She was monitored in the cardiac care unit for 24 hours and later sent to the ward. She was discharged home on day seven with no problems. She had refused a tubal ligation.

## Discussion

Parturients from developing countries often present with undiagnosed or recently worsening symptoms caused by congenital cardiac anomalies. This leads to a higher maternal and infant mortality rate in these countries [5]. Intermarriages also have an impact leading to a higher incidence of such anomalies in these countries. Eisenmenger's syndrome has a mortality rate of 50% to 65% [6] in good centres where high-risk obstetric care is available. Unfortunately many of these women die in early or mid-pregnancy or during delivery due to the unavailability of sophisticated monitoring or expensive drugs. Although no one type of anaesthetic technique has been found to be superior to any other, the use of general anaesthesia (GA) in this case seemed more risky due to the fact that the patient could not lie flat because of orthopnoea. GA can lower the systemic vascular resistance (SVR) severely after induction thereby worsening the right-to-left shunt in such patients, making weaning off the ventilator and extubation at the end of surgery difficult because of the potential worsening of oxygen saturation levels. Regional anaesthesia can also lower the SVR ; however, a slow onset can lower this effect in a predictable manner and thereby reduce the right-to-left shunting. A graded spinal block is a preferable alternative to epidural alone as this can quickly ensure the faster motor block required for surgical intervention and can lessen the time of SVR reduction in total, as compared with an epidural infusion requiring large volumes of medication to achieve the same block. In addition, an epidural catheter can be put in place for post-operative pain relief or in case the surgery is prolonged; additional small boluses of local anaesthetic can also be given to supplement the block intra-operatively in a more graded manner via the catheter if required.

We chose the use of regional anaesthesia (combined spinal epidural) with invasive cardiac monitoring in this case, as well as, administration of nebulisation intra-operatively with PGE1. This has previously been used selectively to reduce shunting in adult respiratory distress syndrome and to improve pulmonary hypertension [7]. Previous case reports and non-randomised studies have indicated that inhalation of aerosolised PGE1 may offer a new life-saving strategy in intractable pulmonary hypertension [8,9]. Its use in the management of parturients has not been described elsewhere in the literature.

## Conclusion

We conclude that the use of nebulised alprostadil following caesarean delivery in a parturient with severe pulmonary hypertension and increased left-to-right shunt can be performed safely, providing a better and more cost-effective outcome.

### Abbreviations

ASD: atrial septal defect; GA: general anaesthesia; PA: pulmonary artery; PAH: pulmonary arterial hypertension; SVR: systemic vascular resistance.

## Competing interests

The authors declare that they have no competing interests.

## Authors' contributions

SS was involved in the conduct of anaesthesia during the case, compiling the data, writing the manuscript and obtaining consent. NL was involved in conduct of anaesthesia and collection of data. Both authors read and approved the final manuscript.

## Consent

Written informed consent was obtained from the patient for publication of this case report. A copy of the written consent is available for review by the Editor-in-Chief of this journal.
